# Pathological effects due to metacercariae of *Clinostomum piscidium* migration in snakeskin gourami (*Trichopodus pectoralis*) in Thailand

**DOI:** 10.3389/fvets.2023.1177218

**Published:** 2023-05-02

**Authors:** Sk Injamamul Islam, Channarong Rodkhum, Piyanan Taweethavonsawat

**Affiliations:** ^1^The International Graduate Program of Veterinary Science and Technology (VST), Department of Veterinary Pathology, Faculty of Veterinary Science, Chulalongkorn University, Bangkok, Thailand; ^2^Department of Veterinary Microbiology and Center of Excellence in Fish Disease, Faculty of Veterinary Science, Chulalongkorn University, Bangkok, Thailand; ^3^Parasitology Unit and Biomarkers in Animal Parasitology Research Group, Department of Pathology, Faculty of Veterinary Science, Chulalongkorn University, Bangkok, Thailand

**Keywords:** aquaculture, digenean trematode, ITS, pathology, Thailand

## Abstract

*Clinostomum* spp. is a fish-borne pathogen and a digenetic trematode with a global range. Despite its zoonotic relevance, the pathogenic impact of the parasite in Thai aquaculture is currently unclear. The present study deals with the pathogenic changes that fluke causes in their host, *Trichopodus pectoralis*, and the molecular confirmation of the *Clinostomum piscidium* by targeting 18 s rDNA and ITS gene. The metacercariae of *C. piscidium* were discovered in the body cavity of infected fish. The gross pathological examination revealed a few white migratory tracks on the surface of the liver and spleen. The migratory track showed histologically as a primary hemorrhage and necrosis of hepatic cells surrounded by a layer of macrophages and epithelioid cells, inflammatory cells, and eosinophilic granular cells in the cytoplasm of liver cells and close to the epithelial cells of the intestine. Also, the migratory track in the spleen appeared as a marked decrease of Red Blood Cell (RBC) count and changes in the necrotic tissue. Infection with this metacercaria produced hepatic tissue injury, which disrupted hepatic metabolism and decreased body weight in the fish hosts. The findings of the study suggest that the pathological effect of *C. piscidium* on farm *T. pectoralis* can cause significant economic loss by stunting fish development and predisposing fish to opportunistic pathogens in the environment. Hence, the treatment and control of *C. piscidium* infections are crucial for the viability of the aquaculture sector since this parasite has been found to cause pathological damage to the vital organs of fish.

## 1. Introduction

The snakeskin gourami (*Trichopodus pectoralis*, Regan 1910; Anabantoidei: Osphronemidae) is one of the most common air-breathing freshwater fishes in the Indo-China Peninsula (1, 2). It is one of the five most significant freshwater species farmed in Thailand (FAO, 2021; DoF Thailand, 2018). However, the rising prevalence of parasitic infections is one of the main challenges to cultivating *T. pectoralis*. In the past, it has been reported that infections caused by *Clinostomum* spp. have been the most important problem with *T. pectoralis* in pond culture, resulting in weight loss and a reduction in growth (3). *Clinostomum* spp. could also serve as a cause of secondary infection for fish species and zoonotic infections for humans (4). Previously, *Clinostomum* spp. has been reported to cause both farmed *T. pectoralis* and humans in Thailand (3, 5, 6).

In most aquatic ecosystems, fishes are hosts to parasites, and sometimes, these parasites can affect fish biology and health (7–10). Some of the most dramatic cases occur when fishes are intermediate hosts for parasites. It has been shown that parasites reduce fish production, affect fish health, make fish more vulnerable to other infections, and even result in fish deaths (11–13). Fish parasites lead to a reduction in economic returns and a decline in the availability of fish as protein-rich sources (14). Therefore, understanding the etiology of parasitic disorders, which influences the choice of possible control, requires accurate identification of parasites down to the genus and species level to apply an effective control or preventive approach.

*Trichopodus pectoralis* is a commercially significant aquaculture species in Thailand, necessitating parasitic research that ultimately leads to sustainable aquaculture management. Despite the commercial importance of farmed *T. pectoralis* in Thailand, the molecular identification of *Clinostomum* spp. and pathological studies have yet to be addressed. Therefore, this study performed molecular identification of *Clinostomum* parasites on *T. pectoralis* culturing central part of Thailand, and by analyzing the pathological parasite effect, this research emphasizes the information on the overall scenario of parasites burden in the farmed *T. pectoralis*.

## 2. Methods

### 2.1. Study area and collection of parasites

The protocols for this research were authorized by the animal ethical requirements of Chulalongkorn University in Thailand, Protocol number: IBC 223103 and IACUC 2231043. Live specimens of 102 *T. pectoralis* were collected from March to November 2022 from aquaculture farms in Samut Prakan (Latitude:13°35′57.6″N and Longitude: 100°35′48.3″E), Samut Songkhram (Latitude: 13° 24′ 52.42″ N and Longitude:100° 00′ 9.50″ E), Samut Sakhon (Latitude:13° 32′ 51.11″ N and Longitude:100° 16′ 25.03″ E), and Kanchanaburi (Latitude: 14°00′14″ N and Longitude: 99°32′53″ E) province in Thailand. The fish were brought to the Parasitology Unit of the Faculty of Veterinary Science, Chulalongkorn University, Thailand, in aerated polyethylene and maintained in aquaria under proper aeration. Tricaine methane sulphonate (250 mg/L) was used to euthanize the fish. A standard protocol was followed for examining the fish for parasites (15). Necropsy was performed, and the exposed body cavity was checked for parasites with the naked eye. After counting the parasites, specific samples were processed for further examination.

### 2.2. Histopathological analysis

Infected liver, spleen, and intestine tissues were properly washed with water before being stored in buffered Formalin for around 24 h for histological examinations. Following thorough washing, the specimens were dehydrated in a succession of Ethyl Alcohols (50, 70, 90, and 100%) and Acetone before being cleared in Methyl Benzoate (16). After dehydration in a graded ethanol series, samples were embedded in paraffin. Paraffin blocks were formed from melted wax kept at 58°C. Histopathology and cellular infiltration at the attachment site were examined by cutting serial sections of 5 μm thickness using the Microtec rotatory microtome (Germany), then staining them with Heidenhain’s Hematoxylin and Eosin. The DPX-mounted sections were examined using an Olympus CX31 light microscope, and the photographs were taken using a Nikon Y-TV55 camera connected to the microscope.

### 2.3. DNA extraction, PCR, and phylogenetic analysis

All the metacercarial specimens were rinsed in saline before being preserved in 70% ethanol until DNA extraction. Genomic DNA from samples was extracted using a Nuleospin^®^ DNA extraction kit (Macherey-Nagel, Düren, Germany), following the manufacturer’s instructions. To amplify 18 s rDNA coding gene region, a set of primer pairs (forward 5′-ATTCCGGAGGGAGCCCTG-3′ and reverse 5′-ATCAACCCAGTCAGCACCC-3′) of 395 product size was designed and used. Similarly, the primer pairs (forward 5’-CACCGCCCTGGCGTAATA-3′ and reverse primer 5′-CGACACTTCGAACGATTTCTAGA-3′) of 801 bp product size were used to amplify the sequences of ITS1-5.8S rDNA-ITS2. To amplify the 18 s rDNA coding and ITS1–5.8 S rDNA-ITS2 non-coding region of the parasites, the primer set was designed by using the Pimer3plus server (17). At first, the newly obtained sequences of the 18 s rDNA gene (Supplementary Table 1) and ITS1–5.8 S rDNA-ITS2 (Supplementary Table 2) gene of *Clinostomum* spp. were aligned with sequences from the Genbank database by MEGA 11. The final comparisons were edited and evaluated by Bioedit v.7.0.5.3 (18). PCR reaction was performed with a total volume of 25 μL, including containing 1 μL of each primer, 4 μL genomic DNA, 12.5 μL 2 × Go Taq^®^ Master Mix (Madison, United States), and 6.5 μL distilled water. The early steps of the PCR cycle condition were carried out with denaturation at 94°C for 5 min; followed by 40 cycles of 94°C for 30 s, annealing at a specific primer at 65/60°C (65°C for 18 s rDNA and 60°C for ITS1-5.8S rDNA-ITS2) for 30 s, and 72°C for 1 min; with a final step of 72°C for 7 min. The PCR products were separated using a 1.5% agarose gel, expected product from PCR was purified using a commercial kit according to the manufacturer’s protocol (NucleoSpin^®^ Gel and PCR Clean-up, Macherey-Nagel, Düren, Germany) and sequenced in both directions by commercial service for DNA sequencing (Celemics, Korea). Sequences were aligned using ClustalW multiple alignments of BioEdit version 7.0.5.3. The aligned sequences were compared with the identity with available sequences in the GenBank database using the Basic Local Alignment Search Tool.^1^ The phylogenetic tree was constructed using the maximum likelihood method with a bootstrap method test of phylogeny with 1,000 bootstrap replications; the nucleotides substitution type and the Poisson substitution model were used, and the pairwise distance homology was computed. MEGA X software program was used to construct the final 18 s rDNA and ITS gene phylogenetic tree (19).

## 3. Results

### 3.1. Collection of parasites and histopathological analysis

**Figure 1:**
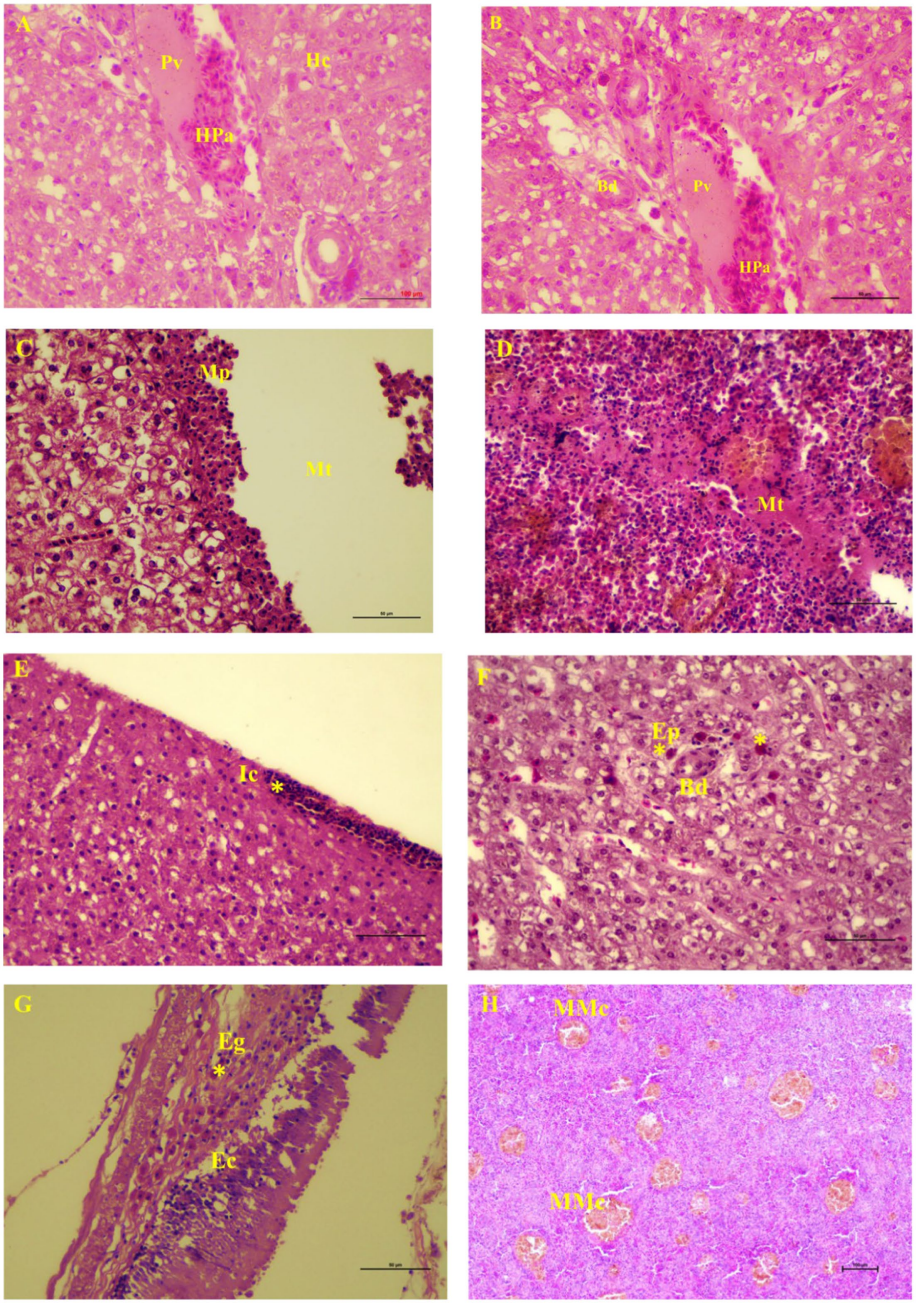
Light micrographs of visceral organs of *Trichopodus pectoralis* non-infected **(A)** and infected **(D–H)** with *Clinostomum piscidium* (H&E stained). **(A)** Liver of non-infected fish shows the normal arrangement of hepatocytes (Hc) into cords or plates. Hepatopancreas (Pa) lies around a portal vein (Pv). **(B)** Normal arrangement of bile duct (Bd) proliferation around the portal vein of the liver cell, **(C)** Liver of infected fish shows migratory track (Mt), macrophages (Mp) near the migratory track, **(D)** Spleen of infected fish shows migratory track where the red blood cells are absent and changes in the necrotic tissue, **(E)** Inflammatory cells (Ic) in the periphery (asterisk) of the liver cells, **(F)** Eosinophilic cells (asterisk) in the cytoplasm around the bile duct of the liver cell, **(G)** Eosinophilic granular cells (Eg; asterisk) near the epithelial cells (Ec) in the infected intestine, and **(H)** Many melano-macrophage cells (MMc) in the infected spleen.

The metacercaria of *C. piscidium* was collected from the body cavity of the fishes, particularly from the surface of the intestine, spleen, and liver (Supplementary Figure 1). In this study, 21 out of the 102 *T. pectoralis* were infected with metacercaria of *C. piscidium*. The overall prevalence of infection was 20.58% and the total number of male and female fish was 41 and 61, respectively. Histologically, the liver was slightly yellowish, and non-infected fish showed a regular arrangement of hepatocytes into cords or plates (Figure 1A). The proliferation of bile ducts in a typical pattern around the portal vein of the liver was noticed (Figure 1B). Metacercariae of *C. piscidium* were found in a non-encysted form, either freely moving or attaching to the adipose tissue, and migratory track of the metacercaria had seen in the liver and spleen of the infected fish (Figures 1C,D). The liver of the non-infected fish showed significant pathological change and revealed hepatopancreases lying around a portal vein (Figure 1A). The liver of *T. pectoralis* harboring metacercariae of *C. piscidium* was marked by the presence of a migratory track and consecutive layers of macrophages and epithelioid cells near the migratory track (Figure 1C). Also, inflammatory cells were noticed in the liver (Figure 1E) followed by eosinophilic cells in the cytoplasm around the bile duct of the liver cell (Figure 1F). The metacercariae of *C. piscidium* adhered to the serosal surface of the intestine, and eosinophilic granular cells near the epithelial cells in the infected intestine were seen (Figure 1G). Several melano-macrophage cells were also noticed in the infected spleen of *T. pectoralis* (Figure 1H).

### 3.2. Molecular identification

**Figure 2:**
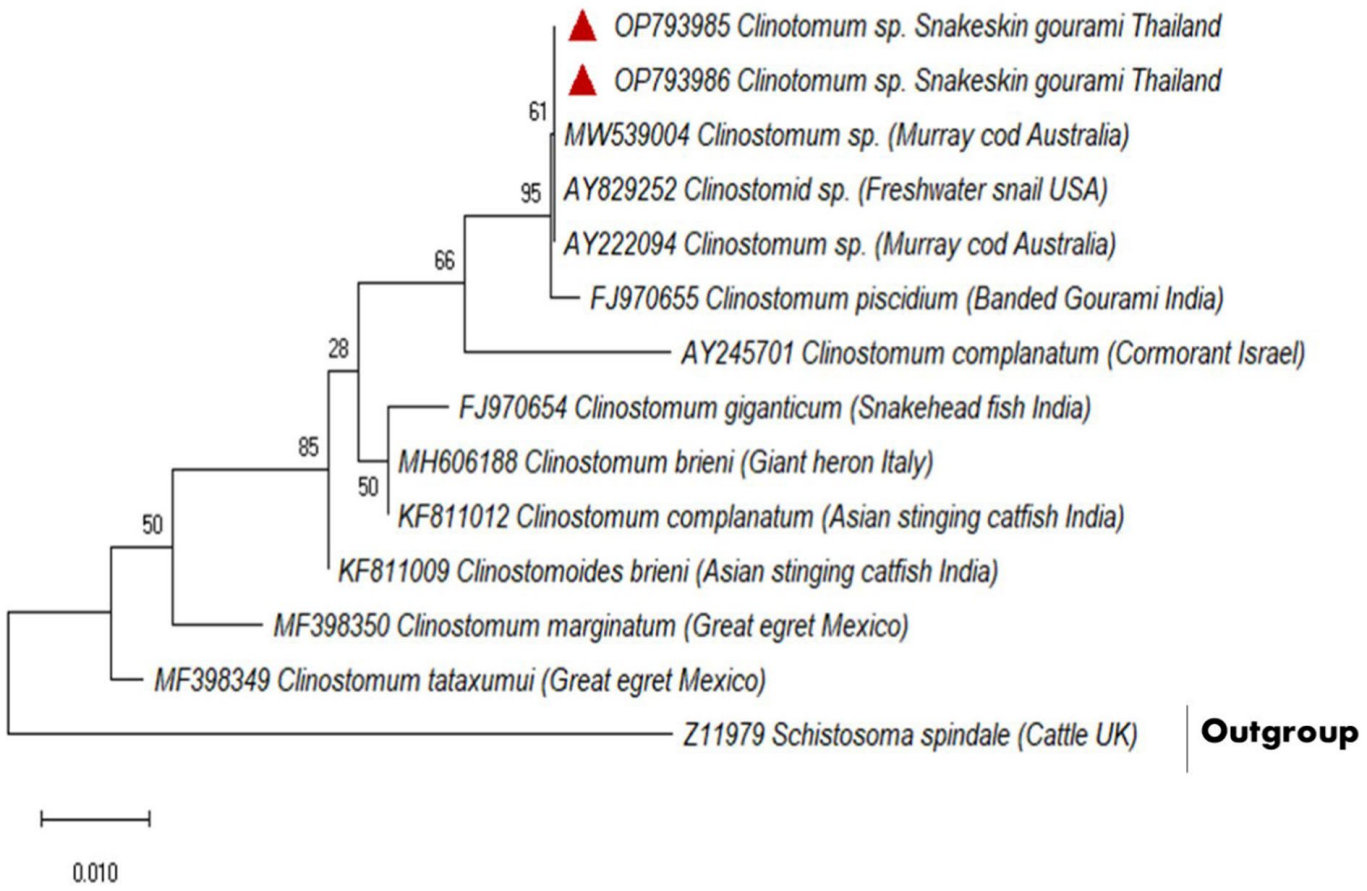
Based on a partial 18S rDNA sequence, the evolutionary relationships between clinostomids were determined using the maximum likelihood method.

A partial fragment of the 18S rDNA gene was generated using PCR amplification. After sequencing, a contig of 395 bp lengths was formed, for which GenBank Accession No. OP793985 and OP793986 were obtained. A phylogenetic tree was created using *Schistosoma spindale* as an outgroup and various clinostomid trematode isolates from diverse geographic locations (Figure 2). The phylogenetic tree indicated that our isolate (OP793985 and OP793986) had 95% similarity with *Clinostomum piscidium* from India (Figure 2; Supplementary Table 3).

**Figure 3:**
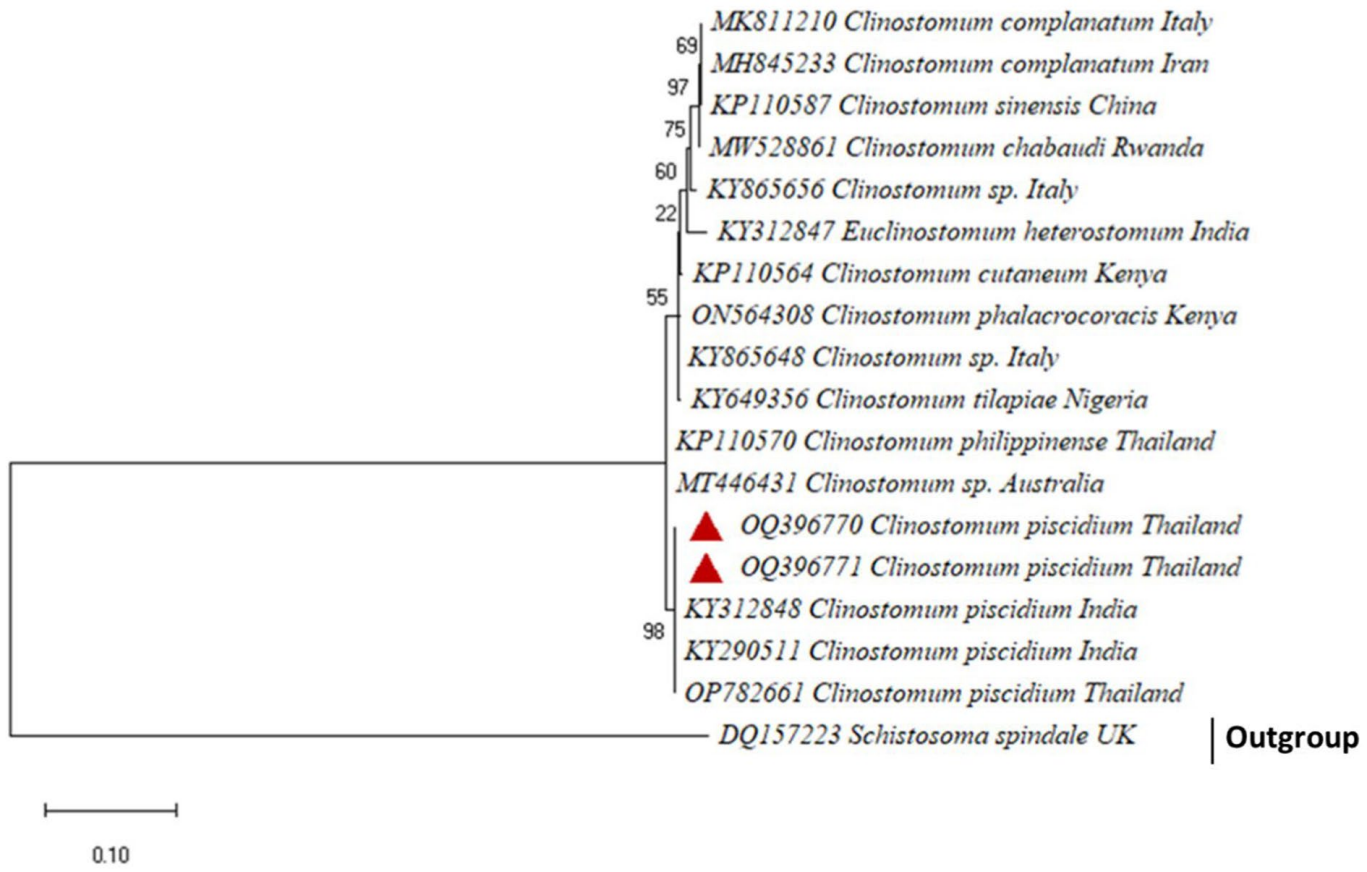
The phylogenetic tree constructed by maximum likelihood displays *Clinostomum piscidium* from *Trichopodus pectoralis* clustering next to the Indian and Thailand Groups of *C. piscidium*.

For the molecular analysis and generating phylogenetic analysis, two accession numbers OQ396770 and OQ396771, an 801 bp fragment of *C. piscidium* ITS1-5.8 S rDNA-ITS2 was uploaded to GenBank. Sequences showing a similarity of 100% to (96–99%) were included in the phylogenetic analysis (Supplementary Table 4). The present partial sequence showed 100% homology with *C. piscidium*, isolated from India and Thailand. The isolated *C. piscidium* from *Colisa fasciata*, *Bubulcus ibis*, and *Trichopodus pectoralis*, and the current species were closely linked in phylogenetic analysis, putting them in the same clade with a high bootstrap value (98; Figure 3).

## 4. Discussion

In the present investigation, severe histopathological damage was observed in the liver, spleen, and intestine of infected *T. pectoralis*, which was marked by the presence of a migratory track produced by the fluke and from host response by chronic inflammation. Granuloma development was revealed by the presence of macrophages and epithelioid cells that had infiltrated the outside rim of the tracks in the liver cells. Similarly, the histopathology of liver damage seen in *Channa punctata* after it had been parasitized with encysted metacercaria of *Euclinostomum heterostomum* revealed a wide area of metacercariae cysts and damaged hepatic tissue (20). Two primary mechanisms could be responsible for the formation of the tracks in the hepatic parenchyma: the first is the mechanical damage caused by the prehensile action of the oral sucker, in conjunction with the mechanical abrasion caused by spines on the fluke’s tegument after they have burrowed and migrated in the liver. In addition, it is possible that the toxic effects of the parasite’s excretory-secretory (ES) products are what caused the damage to the hepatic parenchyma (21). The ES products that are secreted by the metacercaria of Clinostomids have been identified as the cysteine protease that can break down the proteins of the host (22, 23). These products include an enzyme necessary for the progression of the immature flukes’ movement and development. They use these compounds to invade the host tissue and get nutrients for themselves (24). Infection with metacercariae of various digenean is often accompanied by increased eosinophils (25, 26). Adeyemo and Agbede (25) observed eosinophilic granular cell growth in the gills of *Oreochromis niloticus* parasitized with *C. tilapia* metacercariae. In the heart of *Mastacembelus armatus* infected with *Tetracotyle* metacercariae, an increase in the number of eosinophilic granular cells was reported in the region between the host tissue and the parasite (26). In addition, histopathological alterations of *Nandus nandus* parasitized with *C. complanatum* were seen in the liver, characterized by loosening of hepatic tissue, eccentrically located nuclei of hepatocytes, and necrosis (20). Milbourne and Howell (27) suggested that the fluke ES product works similarly to interleukin-5 (IL-5), a cytokine that stimulates myeloid precursor cells, causing differentiation and activation of eosinophils. The current research found that the presence of eosinophilic granular cells in the periphery of the liver reflects the host immune response to the ES antigen on the metacercariae. The infection with metacercariae of *C. piscidium* may compromise the function of the host’s liver and pancreas because of the mechanical damage and toxicity caused by ES products (3). This may occur because ES product causes toxicity. In fish, the liver is considered to be the most important digestive gland since it handles several tasks that are essential to its survival. It substantially impacts the metabolic processes involving carbohydrate, proteins, and lipids, as well as the storage of glycogen. Additionally, this is where the detoxification process takes place (28). Therefore, the massive necrosis of hepatic tissues may be responsible for disturbing the metabolic processes, which may, in turn, disrupt the overall metabolic process of the host, resulting in decreased growth potential.

**Figure 4:**
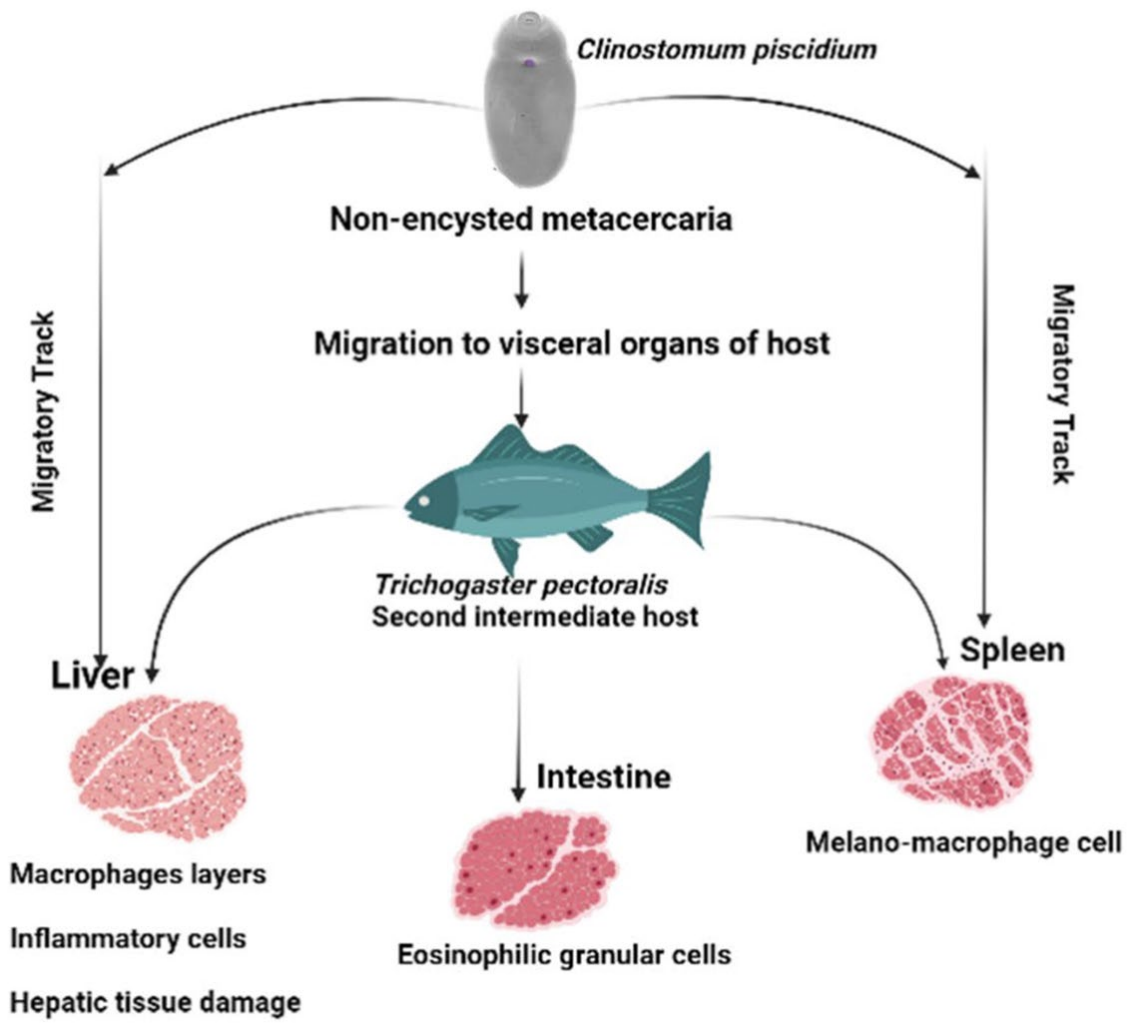
Schematic diagram of the pathological effects of *Clinostomum piscidium* on *Trichogaster pectoralis.*

Melano-macrophages and migratory track were also discovered in the spleen of the infected fish in this study. Increases in pigmented macrophages might be linked to stress and a lack of food (29). To our knowledge this is the first pathological observation of *C. piscidium* in the spleen and intestine of infected fish. In the only previous histopathological report of the attachment sites of *Clinostomum* metacercaria in *T. pectoralis*, an intense hepatic necrosis was found in the liver (3). However, in the species we have studied, this digenea can have significant pathogenic effects on the spleen and intestine, including alterations to necrotic tissue and the presence of eosinophilic granular cells close to the epithelial cells. In addition, the presence of melano-macrophage centers in the infected spleen, can lead to resistant of intracellular bacteria, from which chronic infections may develop (30). A schematic overview of the pathological effect of the *C. piscidium* in the liver, spleen and intestine of the host fish is shown in Figure 4.

Localized hemorrhage caused by *C. piscidium* infection in *T. pectoralis* has been documented in earlier research (3). Fish infected with clinostomatid metacercaria showed retarded development of fish, according to the study (31). Generally, encysted and non-encysted metacercariae of Clinostomids have been distinguished inside the host tissue. The metacercaria of the *C. piscidium* species are the only ones that can survive within the cavity of the second intermediate host without being encysted (3). As a result of their ability to move freely through the visceral organs of the host fish, the non-cysted metacercaria of this species has the potential to inflict far more severe injury to infected fish (32). In the current investigation, *C. piscidium* metacercariae were exclusively collected from the abdominal cavity of infected fish. These provide evidence of the specialized microhabitat that the parasite occupies inside its hosts. Consequently, severe necrosis of hepatic tissues may disrupt metabolic processes, disrupting the fish’s metabolism and stunted development (3).

Furthermore, our data suggest that 18 s rDNA and ITS-1 and ITS-2 sequences are more successful in distinguishing between closely related Clinostomidae taxa (33–35). Figures 2, 3 depicts that our *C. piscidium* isolate from *T. pectoralis* is in the same cluster as an Indian *C. piscidium* isolate from fish and avian hosts.

Overall, Thailand’s commercial aquaculture relies heavily on the *Trichopodus pectoralis*, although the species is very vulnerable to parasite diseases, especially digenean trematode, due to intensive aquaculture practices. *Clinostomum* spp. is an economically important group of parasites among freshwater fishes, snails, and birds throughout the globe. Accurate identification of parasites to the genus and species level is necessary for effective management or preventative measures. Previously, no study had been carried out to establish the molecular identification of *Clinostomum* spp. in *T. pectoralis* in Thailand. It is possible that the pathogenic effects of *C. piscidium* on *T. pectoralis* would inhibit growth and significantly reduce aquaculture production (36, 37). To minimize the economic losses due to *Clinostomum* infection in fish, preventative measures must be implemented *via* the biocontrol of snails, or research on vaccine development and chemotherapy deserves particular attention. The results of this research will help the fisheries community, the government, and other interested parties understand how common *Clinostomum* parasites are in *T. pectoralis* aquaculture and enhance our capacity to recognize parasites, monitor their behavior, connect their expansion to environmental circumstances, and comprehend their contribution to disease etiology. The research covers an essential aspect of food safety and aquaculture sustainability since *T. pectoralis* is a significant source of nutrition for the people in Thailand.

## Data availability statement

The datasets presented in this study can be found in online repositories. The names of the repository/repositories and accession number(s) can be found in the article/Supplementary material.

## Ethics statement

The animal study was reviewed and approved by Chulalongkorn University Animal Care and Use Committee (IACUC 2231043). Written informed consent was obtained from the owners for the participation of their animals in this study.

## Author contributions

SI and PT conceptualized and reviewed the manuscript. SI, CR, and PT collected data and contributed to data interpretation and manuscript preparation. All authors contributed to the article and approved the submitted version.

## Funding

This research project is funded by the Thailand Science Research and Innovation Fund, Chulalongkorn University (FOOD66310019).

## Conflict of interest

The authors declare that the research was conducted in the absence of any commercial or financial relationships that could be construed as a potential conflict of interest.

## Publisher’s note

All claims expressed in this article are solely those of the authors and do not necessarily represent those of their affiliated organizations, or those of the publisher, the editors and the reviewers. Any product that may be evaluated in this article, or claim that may be made by its manufacturer, is not guaranteed or endorsed by the publisher.

## Footnotes

^1^ http://www.ncbi.nlm.nih.gov/BLAST/
