# Supplementary material for: Pathological effects due to metacercariae of Clinostomum piscidium migration in snakeskin gourami (Trichopodus pectoralis) in Thailand
Source: Front Vet Sci. 2023 May 2;10:1177218. doi: 10.3389/fvets.2023.1177218 (PMC10185784; doi:10.3389/fvets.2023.1177218)
Supplement: Supplementary file 1 [file Data_Sheet_1.docx]

Supplementary Material

**Pathological effects due to metacercariae of *Clinostomum pisidium* migration in Snakeskin gourami (*Trichopodus pectoralis*) in Thailand**

**Sk Injamamul Islam, Channorong Rodchum, Piyanan Taweethavonsawat***

Corresponding Author: Piyanan.T@Chula.ac.th

| **Scientific Name** | **Description** | **Accession No.** |
| --- | --- | --- |
| *Clinostomum sp.* | 18S small subunit ribosomal RNA gene | MW539004.1 |
| *Clinostomid sp.* | 18S small subunit ribosomal RNA gene | AY829252.1 |
| *Clinostomum sp.* | 18S small subunit ribosomal RNA gene | AY222094.1 |
| *Clinostomum piscidium* | 18S small subunit ribosomal RNA gene | FJ970655.1 |
| *Clinostomum brieni* | 18S small subunit ribosomal RNA gene | MH606189.1 |
| *Clinostomum complanatum* | 18S small subunit ribosomal RNA gene | KF811012.1 |
| *Clinostomum sinensis* | 18S small subunit ribosomal RNA gene | MK490986.1 |
| *Clinostomum giganticum* | 18S small subunit ribosomal RNA gene | FJ970654.1 |
| *Clinostomum marginatum* | 18S small subunit ribosomal RNA gene | MF398350.1 |

**Supplementary TABLE 1.** List of 18s rDNA gene used for primer design

| **Scientific Name** | **Description** | **Accession No.** |
| --- | --- | --- |
| *Clinostomum piscidium* | internal transcribed spacer 1 and internal transcribed spacer 2 region | KY312848.1 |
| *Clinostomum piscidium* | internal transcribed spacer 1 and internal transcribed spacer 2 region | KY290511.1 |
| *Clinostomum tilapiae* | internal transcribed spacer 1 and internal transcribed spacer 2 region | KY649356.1 |
| *Clinostomum complanatum* | internal transcribed spacer 1 and internal transcribed spacer 2 region | MH845235.1 |
| *Clinostomum complanatum* | internal transcribed spacer 1 and internal transcribed spacer 2 region | MK796829.1 |
| *Clinostomum piscidium* | internal transcribed spacer 1 and internal transcribed spacer 2 region | KY304779.1 |

**Supplementary TABLE 2.** List of ITS1–5.8 S rDNA-ITS2 gene used for primer design.

| **Species Name** | **Accession No.** | **E-value** | **Identity (%)** |
| --- | --- | --- | --- |
| *Clinostomum piscidium* | FJ970655 | 0.00 | 98.93 |
| *Clinostomum brieni* | MH606189 | 0.00 | 98.12 |
| *Clinostomum brieni* | MH606188 | 0.00 | 98.12 |
| *Clinostomum complanatum* | KF811012 | 0.00 | 98.12 |
| *Clinostomum brieni* | MH606187 | 5e-180 | 97.86 |
| *Clinostomum complanatum* | FJ609420 | 6e-179 | 97.59 |
| *Clinostomum complanatum* | AY245701 | 6e-179 | 97.59 |
| *Clinostomum brieni* | KF811009.1 | 2e-178 | 97.59 |
| *Clinostomum giganticum* | FJ970654 | 5e-175 | 97.05 |
| *Clinostomum marginatum* | MF398350 | 1e-171 | 96.51 |
| *Clinostomum tataxumui* | MF398349 | 1e-171 | 96.51 |
| *Clinostomum marginatum* | AY245760 | 1e-171 | 96.51 |
| *Schistosoma spindale* | Z11979 | 3e-137 | 90.74 |

**Supplementary Table 3**. Sequence identity matrix (%) amongst the clinostomids based on the partial sequence of 18S rDNA gene.

| **Species Name** | **Host Species** | **Accession No.** | **E-value** | **Identity (%)** |
| --- | --- | --- | --- | --- |
| *Clinostomum piscidium* | *Colisa fasciata* | KY312848 | 0.00 | 100 |
| *Clinostomum piscidium* | *Bubulcus ibis* | KY290511 | 0.00 | 100 |
| *Clinostomum piscidium* | *Trichopodus pectoralis* | OP782661 | 0.00 | 100 |
| *Clinostomum philippinense* | *Trichogaster microlepis* | KP110570 | 0.00 | 99.52 |
| *Clinostomum sp.* | *Maccullochella peelii* | MT446431 | 0.00 | 99.52 |
| *Clinostomum sp.* | *Amphilius uranoscopus* | KY865648 | 0.00 | 98.39 |
| *Clinostomum tilapiae* | *Synodontis batensoda* | KY649356 | 0.00 | 98.23 |
| *Clinostomum phalacrocoracis* | *Oreochromis niloticus* | ON564308 | 0.00 | 98.07 |
| *Clinostomum cutaneum* | *Ardea cinerea* | KP110564 | 0.00 | 97.91 |
| *Clinostomum sp.* | *Schilbe intermedius* | KY865656 | 0.00 | 97.58 |
| *Euclinostomum heterostomum* | *Channa punctatus* | KY312847 | 0.00 | 97.42 |
| *Clinostomum chabaudi* | *Hyperolius* spp. | MW528861 | 0.00 | 96.94 |
| *Clinostomum complanatum* | *Squalius cephalus* | MK811210 | 0.00 | 96.78 |
| *Clinostomum complanatum* | *Alburnus mossulensis, Capoeta damascina, Garra rufa,* and *Squalius cephalus* | MH845233 | 0.00 | 96.78 |
| *Clinostomum sinensis* | *Ctenopharyngodon idella* | KP110587 | 0.00 | 96.78 |
| *Clinostomum complanatum* | *Oryzias sakaizumii* | LC483163 | 0.00 | 96.62 |

**Supplementary TABLE 4** Sequence identity matrix (%) amongst the clinostomids based on the partial sequence of the ITS gene.


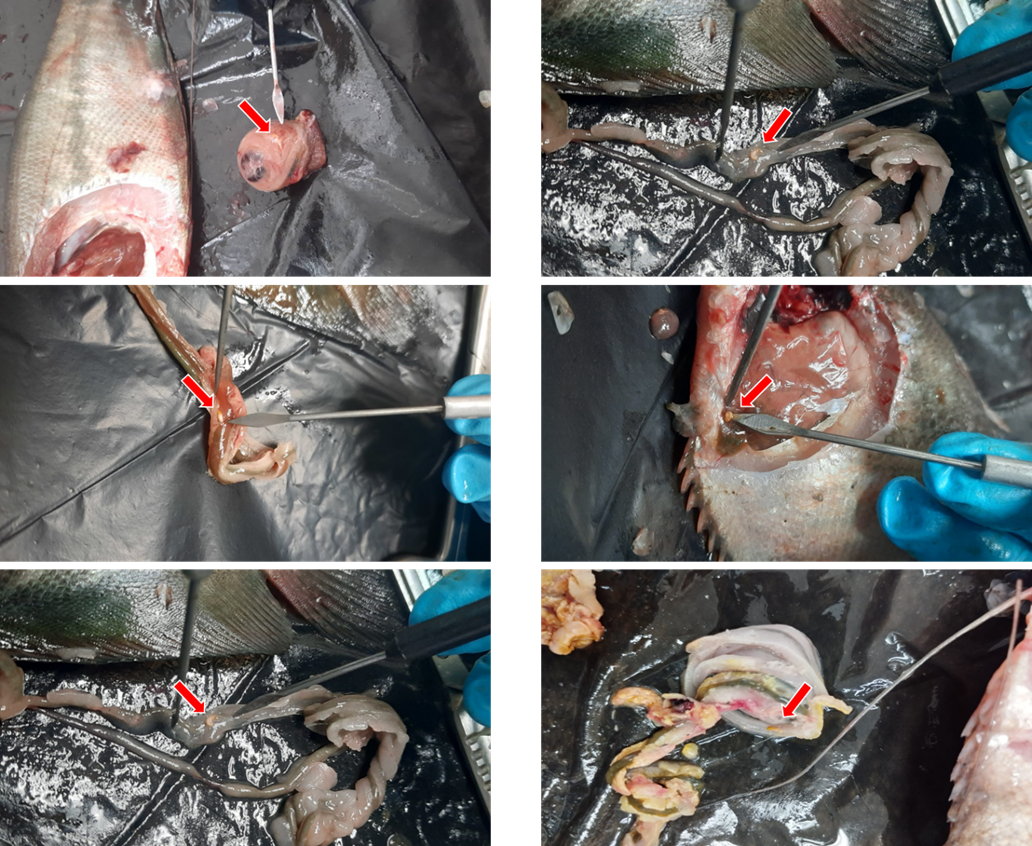


**Supplementary Figure 1**. Collection of the metacercariae of Clinostomum piscidium in different organs of the fish.
